# X-Ray Crystal Structures and Organogelator Properties of *(R)*-9-Hydroxystearic Acid

**DOI:** 10.3390/molecules24152854

**Published:** 2019-08-06

**Authors:** Fioretta Asaro, Carla Boga, Nicola Demitri, Rita De Zorzi, Sara Drioli, Lara Gigli, Gabriele Micheletti, Patrizia Nitti, Ennio Zangrando

**Affiliations:** 1 Department of Chemical and Pharmaceutical Sciences, University of Trieste, via L. Giorgieri 1, 34127 Trieste, Italy; 2 Department of Industrial Chemistry “Toso Montanari”, University of Bologna, viale del Risorgimento 4, 40136 Bologna, Italy; 3 Elettra–Sincrotrone Trieste, S.S. 14 Km 163.5 in Area Science Park, Basovizza, 34149 Trieste, Italy

**Keywords:** organogel, chirality, self-assembly, hydrogen bond, polymorphs, X-ray diffraction, rheology

## Abstract

*(R)*-9-hydroxystearic acid, *(R)*-9-HSA, is a chiral nonracemic hydroxyacid of natural origin possessing interesting properties as an antiproliferative agent against different cancer types. Considering its potential application for medical and pharmaceutical purposes, the structures and rheological properties of *(R)*-9-HSA were investigated. Oscillatory rheology measurements reveal that *(R)*-9-HSA gels only paraffin oil, with less efficiency and thermal stability than its positional isomer *(R)*-12-HSA. Conversely, *(R)*-9-HSA affords crystals from methanol, acetonitrile, and carbon tetrachloride. The single crystal structures obtained both at 293 K and 100 K show non-centrosymmetric twisted carboxylic acid dimers linked at the midchain OHs into long, unidirectional chains of hydrogen bonds, owing to head-tail ordering of the molecules. Synchrotron X-ray powder diffraction experiments, performed on the solids obtained from different solvents, show the occurrence of polymorphism in paraffin oil and through thermal treatment of the solid from methanol.

## 1. Introduction

Plant metabolites are very important renewable resources. The hydroxyacids derived from them are compounds convertible into high-value chemicals [1,2], including bioactive agents with antimicrobial and anticancer activity [3,4]. 9-Hydroxystearic acid (9-HSA) plays an important role in the regulation of cell proliferation and has proven to exert antiproliferative activity toward different cancers [5,6,7]. *(R)*-9-hydroxystearic acid (*(R)*-9-HSA) has proven to be more active than the opposite enantiomer and has been proved to regulate retinal progenitor cell differentiation [8], as well as to act against colon cancer cell proliferation [9]. It is prepared starting from the *(S)*-dimorphecolic acid, a secondary metabolite present in large amounts in the seeds of genus *Dimorphoteca* [10] that is a precious natural source of *(R)*-9-HSA and is otherwise very difficult to obtain in enantiopure form [11]. *(R)*-9-HSA is employed in the preparation of innovative therapeutic nanomaterials [12,13,14].

**Figure 1:**
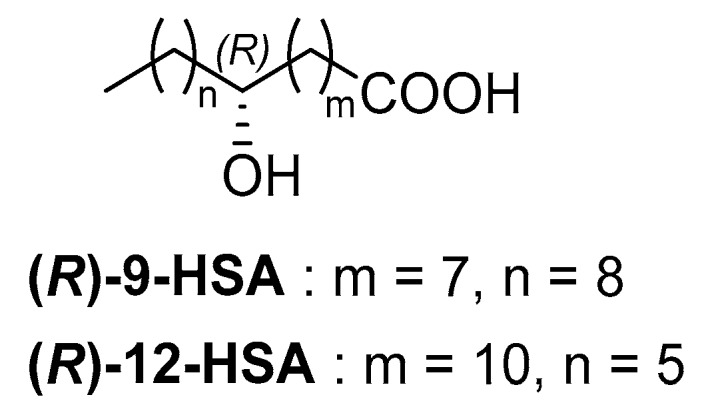
Structures of *(R)*-9-hydroxystearic acid *((R)*-9) and 12-HSA.

*(R)*-9-HSA is a positional isomer of the most prominent example of a naturally occurring hydroxyacid derivative, namely *(R)*-12-HSA (Figure 1), derived from ricinoleic acid, the most abundant fatty acid of castor oil.

*(R)*-12-HSA is a thickener and low molecular weight organogelator [15,16,17] of widespread use in lubricants, paints, and the cosmetic industry. The organogelling ability is shared by HSA positional isomers, with the alcohol OH group sitting on a different carbon of the chain, provided it is at least five bonds apart from the carboxylic carbon [16,18,19]. It is worth investigating *(R)*-9-HSA under this respect, as well as its structural features, in order to gain a deeper knowledge of the supramolecular arrangement of this class of molecules. The structure of *(R)*-12-HSA, both as pure solid and in its organogels, has been the subject of intense structural research. However, its full understanding is hampered by the lack of a single crystal structure, owing to the difficulties in achieving diffraction quality single crystals of molecular gelators of the class of long-chain fatty acids [20,21,22,23]. Structures of two polymorphs of *rac*-12-HSA have been reported [24,25] and their comparison shows in both cases the presence of centrosymmetric carboxylic dimers, made by pairing of the enantiomers. Dimers are connected through hydrogen bonding interactions between the OH group present on the 12^th^ carbon atom of the lipid chain, and the resulting bilayers are further packed into stacks along the *b* crystallographic axis. It must be considered that the structural model of the racemic mixture does not allow to infer the arrangement of the enantiopure compound, and that *(R)*-12-HSA or *(S)*-12-HSA, but not *rac*-12-HAS, display low concentration gelation in organic solvents [26]. Moreover, the structure of a closely related, enantiopure and nongelling species, namely *(R)*-12-HSA methyl ester, consists of a head-tail molecular arrangement imposed by the hydrogen bonding interactions among the OH side groups [27,28]. A similar structural arrangement was suggested in a model of the helical gel fibers of *(R)*-12-HSA [29]. An alternative structural model for the fundamental unit of the organogel fibers, consisting of a twisted dimer, bent at the carboxylic groups linkage, was proposed on the basis of vibrational circular dichroism observations [30]. The relationship between molecular structure and packing is subtle in the case of hydroxyacids, considering that the introduction of only one additional OH in the alkyl tail endows both the molecules and the supramolecular aggregates with chirality. The issue is further complicated by the possible occurrence of polymorphism, a well-known phenomenon with important implications for lipids [31], such as fatty acids and, in particular, the stearic acid [32]. Polymorphs were envisaged also for *(R)*-12-HSA in powders, aerogels, and in organogels depending on the solvent and the thermal history of the specimens [22,33,34], but none currently possesses a thorough structural description. The various forms were discriminated mainly by X-ray scattering techniques, such as WAXS (wide angle X-ray scattering) and SAXS (Small Angle X-ray Scattering). Takeno at al. distinguished four different forms on the base of the long spacing values, ranging from 48.5 to 39.7 Å [22,35]. The alkyl chain packing is a further classification criterion, widely used in the field of lipids, which relies on the XRD short spacing patterns [31,36]. Relevantly, a spacing of ~4.1 Å was reported for the gels of *(R)*-12-HSA in alkanes and thiols in correspondence to a very large d value, whereas ~4.6, 3.9 Å spacings are a distinctive feature of gels in more polar solvents [34].

Here, a multi-technique approach was used to investigate the connection between the mechanical properties and the structure of *(R)*-9-HSA. Analyses included tabletop rheology observations to determine the organogelling capacity in different solvents, oscillatory rheology to mechanically characterize the gel, and circular dichroism (CD) to evaluate the formation of chiral aggregates. In addition, structural analyses were performed using a Synchrotron Radiation (SR) source on the single-crystal and powder samples obtained from solvents with different polarity, and allowed us determine the first high-resolution X-ray structure of an enantiomerically pure hydroxystearic acid.

## 2. Results

### 2.1. (R)-9-HSA Organogel

**Figure 2:**
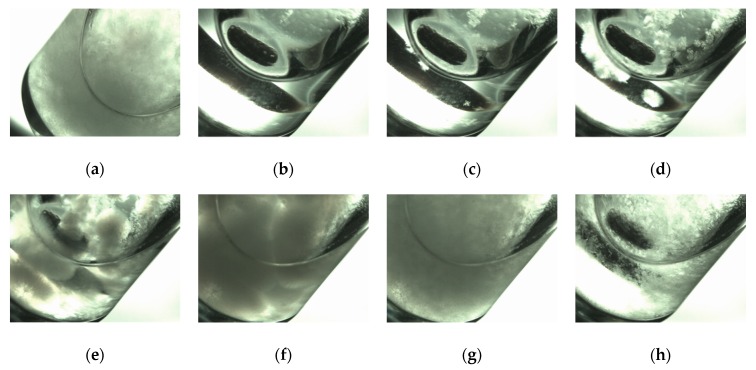
Macroscopic evolution of the solution of *(R)*-9-HSA in acetonitrile (0.5% *w*/*w*): frames taken at successive times. In (**b**)–(**e**) and (**h**) a small stirring bar is detectable at the bottom of the 10-mL test tube.

The organogelling ability of *(R)*-9-HSA was tested in a series of solvents of variable polarity, namely *n*-heptane, *n*-hexane, cyclohexane, carbon tetrachloride, and acetonitrile, in all of which *(R)*-12-HSA is known to form organogels [16]. A fast cooling procedure, in which the hot solution was directly placed at RT, was applied in order to obtain organogels. The attempts failed due to the separation of a solid, with different kinetics depending on the solvent. In the case of acetonitrile, the whole process occurred in the time lapse of about 1 h and was imaged using an optical microscope. As resumed in frames taken at different times (Figure 2) initially a white suspension formed (a), readily dissolving to give a clear solution (b). Then, the molecules started to self-assemble into spherulites (c,d), which grew in number and size (e) until impingement (f). However, they were not stable and progressively converted into small needle-like crystals (g), which precipitated from the solution. Crystal deposition on the bottom of the test tube left clear supernatant solution (h). The crystals were examined by XRD, along with those obtained from carbon tetrachloride (see below).

**Figure 3:**
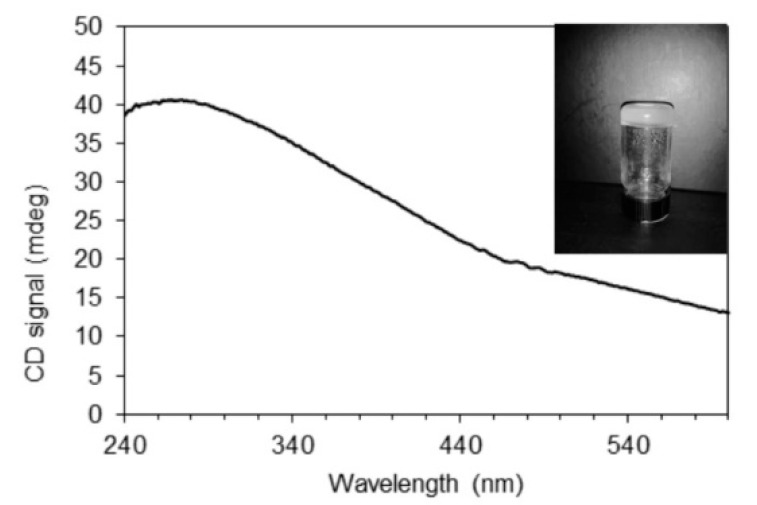
Circular dichroism (CD) spectrum from a layer of the gel. In the inset, the *(R)*-9-HS A–paraffin oil 0.5 % *w*/*w* gel can be seen.

An organogel was obtained exclusively in paraffin oil. A *(R*)-9-HSA concentration as low as 0.5 % *w*/*w* was sufficient to form a gel able to sustain its own weight, as shown in Figure 3. The paraffin oil gel was examined for supramolecular chirality by means of circular dichroism.

The CD spectrum, reported in Figure 3, Figures S4 and S5 displays a broad, positive band at wavelengths longer than those of the electronic absorption of both carboxylic groups and solvent (Figure S3). Therefore, it must be attributed to selective reflection of left-handed circularly polarized light by the left-handed helical arrangement of *(R)*-9-HSA [33], with pitch on the order of the wavelength of the maximum, at about 270 nm. This phenomenon is well-known for the helical fibers present in the organogels of enantiopure 12-HSA, usually occurring with negative CD curves for the *R* enantiomer and positive for the *S* enantiomer [33]. However, the relationship between molecular chirality and supramolecular helical handedness is far from straightforward. As for the supramolecular helix formed by both thermotropic and lyotropic cholesteric liquid crystals, for which the handedness depends on temperature and solvent [37], as well as in the case of 12-HSA and its soaps, helices with different handedness were observed [21,38]. For comparison purposes, we acquired the CD spectra from *(R)*-12-HSA gels in paraffin oil, as well as in cyclohexane, *n*-hexane, and *n*-heptane (Figures S4, S6 and S7). For the gels prepared in paraffin oil, the CD signal is positive. For both *(R)*-12-HSA and *(R)*-9-HSA, the CD signal somewhat shifted to shorter wavelengths for the former. On the contrary, *(R)*-12-HSA gels obtained in cyclohexane, *n*-hexane, and *n*-heptane show a negative CD signal, as reported in literature in the case of the *(R)*-12-HSA gel in aromatic solvents [33].

### 2.2. Rheological Measurements

The comparison between the paraffin oil organogels was extended to the mechanical properties by means of rheological measurements.

Samples used for rheological measurements were prepared by heating a 1% *w*/*w* suspension of oranogelator in paraffin oil in a microwave reactor. The use of the microwave reactor is advantageous because it allows a clean, fast, and above all, highly reproducible preparation of thermoreversible organogels. Moreover, owing to the pressure control device, it makes it possible to operate safely at temperatures close to the gelator melting temperature, commonly required for dissolution, as well as with low boiling solvents.

The rheological measurements were performed both on freshly prepared samples and on aged samples to investigate their time stability.

**Figure 4:**
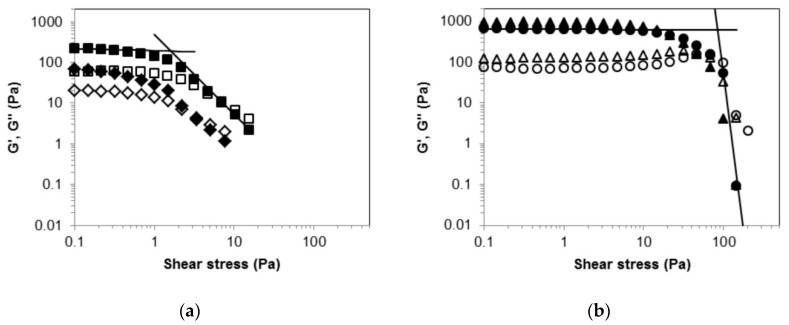
Amplitude sweep tests. Storage modulus, G′, (filled symbols) and loss modulus, G″, (empty symbols) as functions of stress amplitude at an oscillation frequency of 1 Hz for 1% *w*/*w* gels in paraffin oil: (**a**) Freshly prepared *(R)*-9-HSA gel (squares) and the same gel after eight days of aging at 25 °C (diamonds); (**b**) freshly prepared *(R)*-12-HSA gel (circles) and the same gel after eight days of aging at 25 °C (triangles).

Large amplitude oscillatory stress measurements were performed in order to check the material mechanical behavior outside the linearity region [39]. For the *(R)*-9-HSA gel sample, at low stress amplitude the elastic modulus, G’ = 216 Pa, is higher than the viscous modulus, G’’ = 60 Pa, (Figure 4a) with a solid-like behavior.

Upon increasing stress amplitude, the material exhibits a relatively smooth yielding transition from a solid to a shear thinning liquid. The determination of the yield stress, σ_y_, and even its definition are subjects of intense debate [40]. Nevertheless, fair estimates of σ_y_ values can be obtained from oscillatory measurements [41]. Here, we consider as σ_y_ the stress value at the intersection of the two tangent lines to G’ data plotted vs. σ, in the region of lowest stress amplitude and in the flow region, respectively [41,42], as depicted in Figure 4. They are power laws best interpolations, which appear as straight lines in the log-log plots. The σ_y_ value obtained for the *(R)*-9-HSA sample is 2 Pa at G’ = G’’ = 191 Pa, whereas the critical stress values, σ_c_, which corresponds to the intersection of the G’ and G’’ curves [42], occurs at 7 Pa with critical modulus G’ = G’’ = 12 Pa.

The G’, G’’, σ_y_, and σ_c_ values are significantly reduced after eight days (Figure 4a), indicating that *(R)*-9-HSA gel is not stable over time. In the eight-day aged sample, some solid separating from the gel was observed.

Similar rheological tests were carried out on the corresponding gel of *(R)*-12-HSA. They revealed that the *(R)*-12-HSA gel is both more rigid and stronger, with G’ and G’’ values of 655 Pa and 75 Pa, respectively. σ_y_ is 84 Pa with G’ = G’’ = 619 Pa and σ_c_ is 72 Pa with G’ = G’’ = 141 Pa. Such values are remarkably higher than those of the *(R)*-9-HSA gel. Moreover, the *(R)*-12-HSA gel is stable over time, as proved by the small differences between the datapoints of the fresh and aged samples (Figure 4b).

**Figure 5:**
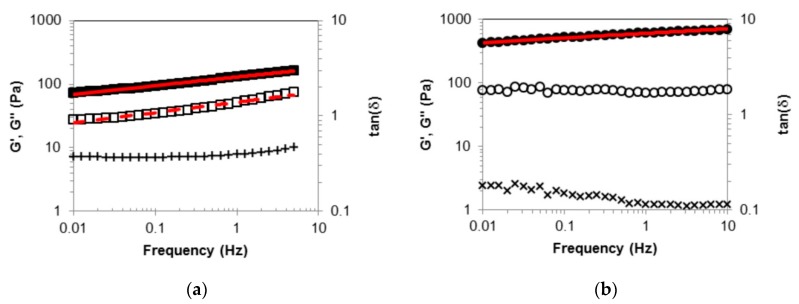
Dynamic moduli, G′ (filled symbols) and G″ (empty symbols), and loss tangent (crosses) versus the frequency of the oscillatory stress for the freshly prepared 1% *w*/*w* gels in paraffin oil: (**a**) *(R)*-9-HSA; (**b**) *(R)*-12-HSA. The red solid lines are the power fits for G’ data and the red dashed line for G’’ data.

The frequency sweep experiment carried out on both gels are reported in Figure 5.

For the *(R)*-9-HSA gel, in the sampled frequency range of 0.01–5 Hz, G’ is higher than G’’. The dynamic moduli appear almost parallel in the double logarithmic plot and nicely fitted to power laws of the kind G_0_∙ν^n^, with exponents assuming close values for G’ and G’’, i.e., n = 0.13 and 0.16, respectively, with G_0′_ = 129 Pa and G_0′_’ = 52 Pa. The loss tangent, tan(δ), which corresponds to the G’’/G’ ratio, has a constant value of about 0.4. Thus the *(R)*-9-HSA sample, despite being a viscoelastic material with prevalent elastic character, cannot be identified as “gel” according to the common criterion that G’ values are much higher than G’’ for gels [43].

The experimental outcome of oscillatory tests performed on the *(R)*-12-HSA gel is in fair agreement with those reported in literature for 12-HSA organogels in paraffin oil and high alkanes [44,45,46], considering that paraffin oil is a variable mixture of hydrocarbons. As shown in Figure 5, the rheological response of the *(R)*-12-HSA gel sample differs quantitatively, not qualitatively, from that of the *(R)*-9-HSA gel. The power fit to G’ returns the equation 606∙ν^0.7^, while G’’ is almost independent of frequency. The loss tangent value, slightly decreasing with frequency, is on the order of 0.1, indicating that the *(R)*-12-HSA one can be categorized as a true gel according to the conventional criterion, recalled above.

### 2.3. X-Ray Single Crystal Structure

**Figure 6:**
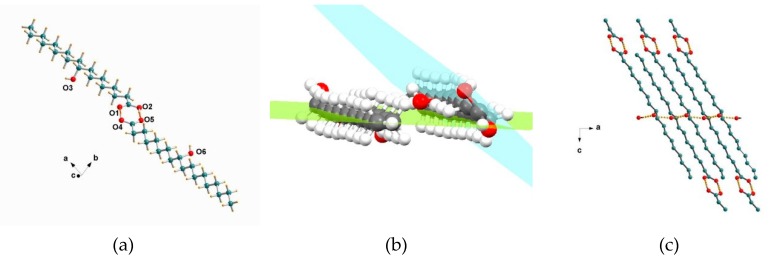
Crystal structure of *(R)*-9-HSA crystallized from methanol: (**a**) the *(R)*-9-HSA dimer, crystallographic independent unit, with molecule **1** on the left side and molecule **2** on the right side. (**b**) Average planes across the carbon atoms of the alkyl chains of the dimer. The dihedral angle is 38.8°. (**c**) Head-tail packing.

Crystals of *(R)*-9-HSA suitable for single-crystal analysis were obtained from a methanol solution. X-ray diffraction datasets were collected at RT and at 100 K. The small differences between cell parameters (Table S1) of the two structures can be attributed to thermal expansion, but overall, the data rule out a structural transition due to a temperature change, the structures being isomorphous. The crystals of *(R)*-9-HSA in methanol belong to the non-centrosymmetric triclinic space group (*P*1). The asymmetric unit consists in a dimer, with a 46.8 Å distance between the two extreme carbons. The two independent molecules, later referred to as **1** and **2**, are depicted in Figure 6a. Molecules **1** and **2** are rotated with respect to each other, with average planes of each alkyl chain forming a dihedral angle of about 40° (Figure 6b).

There is a notable twist of the carboxylic groups with respect to the average planes of carbon atoms of each chain, with a related strain in the crystal lattice. The C-C-C-O torsion angles are −21.0 (3), 160.4 (2)° for molecule **1** and 41.3 (3), −141.2 (2)° for **2**, as reported in Table 1 and Table S2.

**Figure 7:**
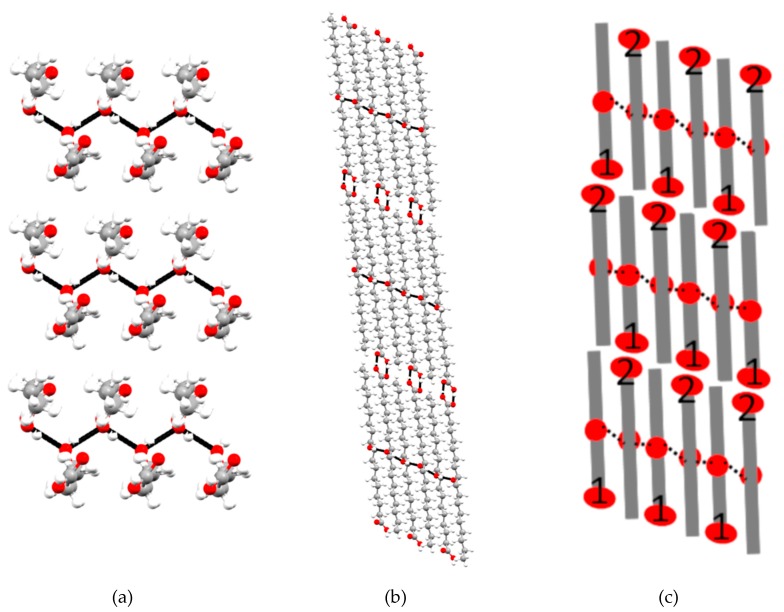
Crystal packing in the structure of *(R)*-9-HSA crystallized from methanol: (**a**) View parallel to the layer planes, with thick black lines highlighting hydrogen bonds between the OH groups placed halfway the alkyl chains. (**b**) View of one-layer plane, perpendicular to (**a**). Black lines indicate all hydrogen bonding interactions present in the structure, among the OH group placed halfway the alkyl chains and among the carboxylic head groups. (**c**) Schematic representation of a layer in the molecules of *(R)*-9-HSA, in the same direction as (**b**). Heads are depicted as red ovals, tails as grey rectangles, and hydroxyl groups as red circles, while dashes represent hydrogen bonding interactions.

The dimers elongate along the 101 crystal direction, with the average axis formed by their carbon atoms tilted by about 60° with respect to the crystallographic axis *a*, are head-tail oriented (Figure 6c). Such an arrangement favors the formation of a hydrogen bond series between the OH groups placed halfway the alkyl chains. Each molecule **1** forms two hydrogen bonds with neighboring molecules **2** of a different layer, parallel to the layer of molecules **1** and 4.5 Å apart (Figure 7a). This arrangement allows the formation of an undulated and interconnected layer parallel to the *ac* crystallographic plane as shown in Figure 7b,c.

The high resolution of the present structure allows an accurate measurement of distances between oxygen atoms forming the bridging hydrogen bonds, reported in Table 2 and Table S3. Noteworthy, OH dipoles point in the same direction, likely yielding a resultant electric dipole moment along the crystallographic axis *a*. Each OH group acts as a hydrogen bond donor and acceptor at the same time, with O…O distances of O(3)-H…O(6) = 2.753(3) and O(6)-H…O(3) = 2.743(3) Å (Table 2).

### 2.4. X-Ray Powder Diffraction

**Figure 8:**
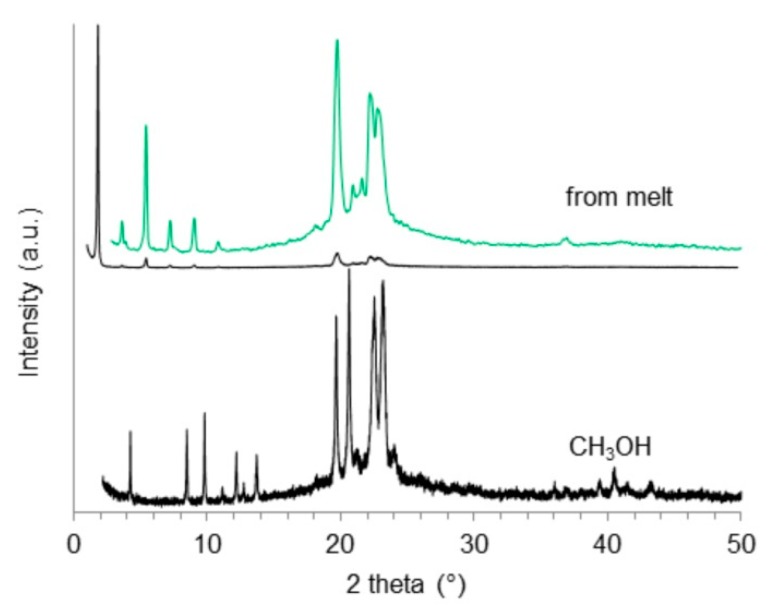
Experimental powder patterns for *(R)*-9-HSA crystallized from CH_3_OH (bottom trace) and for the solid obtained after recrystallization from melt (upper trace, black line, with the 5 × magnification of the 2.8–50° region, in green).

X-ray powder diffraction experiments were used to investigate the formation of different polymorphs in samples of *(R)*-9-HSA crystallized from solvents with decreasing polarity (CH_3_OH > CH_3_CN > CCl_4_). Diffraction patterns are presented in Figure 8 and Figure S9. Data were collected using synchrotron radiation at the MCX beamline of the synchrotron Elettra (Basovizza, Italy).

A whole powder pattern fitting (Pawley method with TOPAS V5 [47]) on the experimental data was performed in *P*1 space group starting from the unit cell parameters refined for the single crystal of *(R)*-9-HSA, crystallized from methanol. For comparison, the simulated powder pattern of the structure obtained from single crystal is reported in Figure S8. Fittings resulted in a good R_wp_ of 4.61%, 5.6%, and 5.7% for diffraction patterns recorded on powder samples obtained from methanol, acetonitrile, and carbon tetrachloride, respectively. The refined unit cell parameters of the three samples are reported in Table 3. These results clearly indicate that the three crystalline phases are similar, and are isomorphous with the single crystals obtained from methanol.

XRPD data were also obtained for a sample crystallized from a paraffin oil aged gel and a sample crystallized from melt (Figure 8 and Figure S10). The comparison between the XRPD patterns of the sample obtained in methanol and the one that underwent the thermal melting and recrystallization (Figure 8) clearly indicates that the two crystalline phases are different, proving the presence of a polymorphic variety of *(R)*-9-HSA. The polymorph obtained after thermal treatment shows similarities with the crystalline powder obtained from paraffin oil (Figure S10). Due to the presence of the paraffin oil (hump around 20°), the signal to noise ratio in the pattern obtained for the crystalline powder in this solvent is very low. Indexing was obtained by comparison to the pattern of the sample crystallized from melt (Figure 8).

The XRD pattern of the thermally treated sample of Figure 8 is characterized by a very strong reflection at small angle (1.9°), corresponding to d= 49 Å. This value exceeds the spacing (d = 21 Å) of the compound crystallized from methanol (Figure 8), suggesting a loss of order in the layers packing direction, doubling the translational vector of the crystal.

The presence of a small number of peaks, together with the low symmetry of the space group, made it very challenging to index the pattern obtained from crystals recrystallized from melt. Among the several possible solutions identified, the triclinic unit cell *P*1 was chosen, considering that enantiomerically pure compounds only pack in acentric space groups. The structure of this polymorph of *(R)*-9-HSA was solved by a simulated annealing protocol, starting from the *(R)*-9-HSA single crystal model (see Section 4.7 for details).

The crystallographically independent unit in the structure of the thermally treated sample is a bent twisted dimer, as depicted in Figure S11a. The twist angle between the average planes of the alkyl chains is 90°. Unlike the structure of the first polymorph of *(R)*-9-HSA (Figure 6b) and the structure of *(R)*-12-HSA methyl ester, in the thermally treated sample, alkyl chains are packed in a parallel fashion (Figure S11b,c). Oxygen atoms of OH groups of adjacent side chains are oriented in different directions and the different packing of the chains compared to the first polymorph of *(R)*-9-HSA causes an increase of the distance between oxygen atoms of adjacent carboxylic groups to about 4 Å, suited for a weak hydrogen bonding interaction. The similarities between the dimer observed in this structure and the one for *(R)*-12-HSA in benzene organogel, proposed by Sato et al. on the basis of vibrational dichroism observations and theoretical calculations [30], are undoubtedly noteworthy and support the present structural model.

## 3. Discussion

The availability of a single-crystal high-resolution X-ray diffraction structure of *(R)*-9-HSA provides a fundamental contribution to our understanding of the self-assembly of these chiral nonracemic molecules. The arrangement of *(R)*-9-HSA molecules in solids obtained from methanol, acetonitrile, and carbon tetrachloride is similar to that found in the crystal of the methyl ester of *(R)*-12-HSA, thus supporting the importance of hydrogen bonds in carbinols with unique configuration. The presence of these intermolecular interactions compels the molecules to lie head-tail in order to maximize the strength of interaction [28]. The hydrogen bonds of *(R)*-9-HSA crystallized from methanol and of *(R)*-12-HSA methyl ester have similar donor-to-acceptor distances, shown in Table 2, Tables S3, and S4. The most remarkable differences between the structures of the two enantiomerically pure compounds are due to conformational distortions, the tilt of the chains for the *(R)*-9-HSA, and a different arrangement of layers of molecules held together by hydrogen bonding interactions, leading to a different packing and to different translational vectors in the crystal structure (vide infra).

A significant difference in the hydrogen bonding interaction pattern can be inferred in the crystal of *(R)*-9-HSA crystallized from melted. The structure, obtained through the combination of experimental XRDP data and simulated annealing, suggests the loss of the hydrogen bond interactions between side chain hydroxyl groups and a weakening of the interaction between carboxylic head groups (see Results).

**Figure 9:**
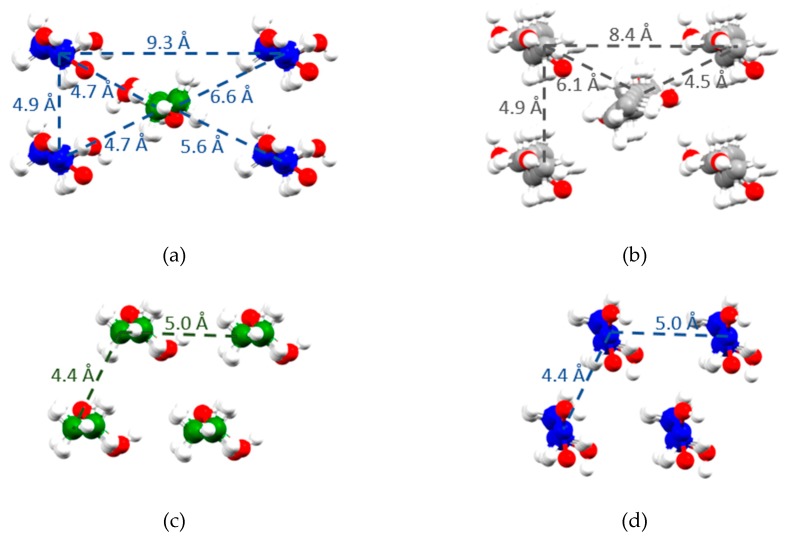
Packing of the lipid chains in the structures of (**a**) *(R)*-9-HSA crystallized from methanol, (**b**) *(R)*-12-HSA methyl ester [28], and (**c**,**d**) two crystallographically independent layers in the structure of *(R)*-9-HSA crystallized from melt. Different colors represent crystallographically independent molecules disposed in opposite directions. Dashed lines show distances between the carbon of the alkyl chain bearing the OH moiety, i.e., carbon 9 in panels (**a**), (**c**), and (**d**), and carbon 12 in panel (**b**).

An important difference between the polymorphs of *(R)*-9-HSA regards the packing of the alkyl chains. In the structure of *(R)*-9-HSA crystallized from methanol, alkyl chains are disposed in a head-tail packing, where every chain is surrounded by four chains of the other crystallographically independent molecule present in the unit cell, disposed in the opposite direction (Figure 9a). Considering only molecules disposed in the same direction (blue molecules in Figure 9a), the packing is approximately rectangular. Such arrangement is interdigitated by molecules disposed in the opposite direction (green in Figure 9a). The arrangement closely resembles that of the crystal structure of *(R)*-12-HSA methyl ester (Figure 9b), where, however, the disposition of parallel chains is exactly rectangular, as imposed by the monoclinic crystal symmetry. In addition, in this structure, the crystallographic symmetry axis *2_1_* relates molecules pointing in opposite directions. Interestingly, distances between parallel and antiparallel alkyl chains are similar in the structure of *(R)*-9-HSA polymorph crystallized in methanol and in the structure of the *(R)*-12-HSA methyl ester, as highlighted in Figure 9a,b.

The *(R)*-9-HSA polymorph obtained from melt shows a completely different packing of the alkyl chains (Figure 9c,d). In this structure, alkyl chains are disposed in the same direction as the neighboring molecules, forming a layer of parallel molecules. In the crystal, layers of molecules pointing in opposite directions are stack on top of each other (Figure S11). Distances between the alkyl chains are shorter than in the structure of the other polymorph of *(R)*-9-HSA or in the structure of the *(R)*-12-HSA methyl ester.

These differences might provide clues regarding the gelling ability of chiral nonracemic hydroxyalkanoic acids, which is not shared by their esters. Despite the fact that *(R)*-9-HAS is not able to induce gelation in methanol, its structure from methanol displays many analogies with the structural models proposed for the helical fibers of the *(R)*-12-HSA organogel network. The observed interdigitated packing complies with the prediction of Abraham et al. [18]. One-dimensional unidirectional interactions have been recognized as a key feature for preferential unidirectional growth into fibers of molecular gelators [48,49]. In the crystals obtained from solvents (CH_3_OH, CCl_4_, or CH_3_CN), such interaction can be identified with the infinite chain of unidirectional hydrogen bonds, which are strengthened by σ-cooperativity [50]. Relevantly, the 1D hydrogen bonding interactions are present in the crystals from solvents but not from melt. The vertical cross-section of the gel fiber might be similar to the image of Figure 6c, with the chains of hydrogen bonds likely running along the direction of the long axis of the fiber [15].

The structure of *(R)*-9-HSA from methanol noticeably differs from the structures of the racemic mixture of 12-HSA, which correspond to stacks of cyclically racemic pairs held together by hydrogen bonds and devoid of stress, with coplanar alkyl tails and additional hydrogen bonds involving the side chain OH groups confined within each layer [24,25].

Concerning the supramolecular chirality of the gel aggregates of *(R)*-12-HSA organogels, Laupheimer et al. [44] highlighted the striking similarity between the structural motifs in *(R)*-12-HSA organogels, imaged in beautiful freeze-fracture electron micrographs, and the helical nanofilament liquid crystalline phase (B4), formed by achiral molecules possessing a bent core and a dipole moment. In that case, the molecules are tilted and arranged into layers which are frustrated, owing to the mutual orthogonal tilt direction of the two flexible arms placed at both ends of the bent core. Frustration is relieved through saddle splay curvature of the layers which, combined with the polarization of the self-assemblies, leads to a twisting deformation of the fibers [51]. Thus, a bent *(R)*-12-HSA dimer, of the kind already suggested for benzene organogels [30], was also considered as the building unit for the networks of *(R)*-12-HSA gels in *n*-decane [44]. This alternative model recalls the packing mode of *(R)*-9-HSA solid obtained in paraffin oil and after the thermal treatment from XRDP data (Figure S11).

The connection between the structural polymorphism and the morphology of the self-assemblies is a delicate issue. It must be further investigated whether the spherulite structure (Figure 2) corresponds to the same polymorph of the needle-like crystals obtained from methanol, carbon tetrachloride, and acetonitrile (Figure 8 and Figure S9), to a transient phase similar to the one observed in paraffin oil or after thermal treatment (Figure 8 and Figure S10), or whether it is a different polymorphic form altogether. The formation of spherulites and their successive conversion into needle-like crystals was reported in literature for *(R)*-12-HSA in alcohol solutions, whose spherulites could be stabilized for a longer time by Nujol [33].

Though *(R)*-9-HSA shows poor organogelator capacity, gelling exclusively in paraffin, the analogies in the mechanical behavior indicate that the softer and weaker *(R)*-9-HSA paraffin oil gel is a soft material of the same nature of the stronger *(R)*-12-HSA gel. The rheological behavioral pattern of *(R)*-12-HSA in various solvents, such as dodecane, toluene, and nitrobenzene [45], suggests that its organogels may be considered soft glassy materials [52], characterized by structural disorder and metastability.

We speculate that the different behavior of *(R)*-9-HSA in paraffin compared to other solvents might be the result of kinetics rather than structural stability, and that similar interactions between molecules of *(R)*-9-HSA are responsible for both the formation of the gel network in paraffin and the formation of crystals in other solvents. However, while the former is entrapped in a kinetic metastable state (gel), the latter quickly evolves in crystals. This behavior is consistent with a stronger tendency of *(R)*-9-HSA to crystallize compared with *(R)*-12-HSA. As a matter of fact, gels are an intermediate situation between solution and macroscopic phase separation, and they rely on the interplay of mutual intermolecular interactions between gelator and solvent. It appears that the interactions of *(R)*-9-HSA with most organic solvents are too weak to form stable gels. Intermolecular interactions in solution [53] and in gels [54] were evaluated by means of the Hansen solubility parameters (HSPs) that distinguish three main contributions: dispersive interactions (δ_d_), polar interactions (δ_p_), and hydrogen bonds (δ_h_) [53]. By the scrutiny of a very large number of solvents, Gao et al. [16] assessed both the HSPs of *(R)*-12-HSA and, with great precision, the range of HSPs of the organic solvents suited to organogelation, highlighting the role of both weak polar interactions and, above all, hydrogen bonds [16]. We argue that, in order to yield a gel, *(R)*-9-HSA requires solvents with a different δ_p_, δ_h_ balance with respect to *(R)*-12-HSA, owing to the enhanced cohesion in the solid state. However, when comparing such structurally close molecules, the main factors to take into account are the specific molecular interactions, while bulk solvent parameters probably are not detailed enough to be predictive.

The transition between the supramolecular chiral aggregates, made of chiral building blocks, and microcrystals, can be phenomenologically accounted for in terms of Landau free energy, in analogy to the possible mechanism identified for a similar phenomenon involving amyloidogenic polypeptide systems [55]. After all, coexisting microcrystals and twisted fibers were detected also in *(R)*-12-HSA organogels [18]. The free energy dependence on the twist angle between building blocks, φ has a minimum in correspondence of the fiber characteristic φ. For specific material parameters, the depth of this energy minimum progressively decreases upon fiber thickening to a critical width, at which the crystal (φ = 0) is the only stable form. The coefficients involved in the expansion of the Landau free energy as a series of powers of φ are expressions of the material properties, namely the bulk crystal and the fiber elastic parameters, affected by solvent, and of φ, the intrinsic twist that best accommodates the individual units with defined chirality. The constant term is relevant to the unfavorable interaction between fiber surface and solvent and the crystal is favored by far when the interaction with solvent at the interface exceeds the twisting transition energy. This theory is particularly interesting for HSA organogels, as the role of the solvent is of paramount importance.

## 4. Materials and Methods

### 4.1. Synthesis of (R)-9-Hydroxystearic Acid

The *(R)*-9-HSA was obtained as previously reported [9] and then crystallized from methanol, M.p. 85 °C by DSC measurements performed on a Q2000 (TA instruments, New Castle, DE, USA), with a temperature ramp of 5 °C/min (see Figure S1).

### 4.2. High-Temperature Treatment of (R)-9-Hydroxystearic Acid

A small amount of the crystalline sample of *(R)*-9-HSA from methanol, placed in a 0.5-mL polypropylene PCR microcentrifuge tube, was heated to 90 °C and then slowly cooled down by 2° every 30 min to 65 °C in a Techne 3PrimeG thermocycler (Techne, Stone, Staffordshire, UK). Afterwards, the sample was left to freely reach room temperature (RT).

### 4.3. Gel Preparation

The suspension of organogelator (up to 1% *w*/*w*) and solvent was heated in a water bath for 20 min with stirring, then it was left to cool without stirring. Various solvents were tested, namely cyclohexane, *n*-hexane, *n*-heptane, and paraffin oil. The gel was obtained exclusively in paraffin oil. In the other cases, precipitates were observed. All the solvents were from Sigma Aldrich, except paraffin oil for IR with viscosity (20 °C) 110–125 mPa∙s, Fluka.

The gel samples for rheology measurements were prepared by microwave heating of the mixture (*R*)-9-HSA-paraffin oil (1% *w*/*w*) at 90 °C for 10 min under magnetic stirring in a CEM Discovery reactor. Then, samples were left to cool down inside the reactor after stirring was stopped.

The microwave procedure was repeated with acetonitrile (1% *w*/*w*) and carbon tetrachloride (0.5% *w/w*), but the outcomes were solids which separated from the solvent. The formation of the precipitate on cooling in acetonitrile was followed in the 10-mL microwave test tube by a Stereozoom Classmag 51 Trino Motic optical microscope equipped with a Moticam 2 digital camera (Motic, Kowloon, Hong Kong).

The gel samples of (*R*)-12-HSA (12-HSA 95% Alfa Aesar) in paraffin oil for comparison purposes were prepared by the same procedure.

### 4.4. Circular Dichroism Measurements

The circular dichroism (CD) measurements were carried out on a JASCO J-710 spectropolarimeter (Jasco, Easton, MD, USA) equipped with a rectangular, 1-cm optical path, quartz cell. Owing to the turbidity of the sample, the CD spectrum was recorded for a layer of *(R)*-9-HSA 0.5% *w*/*w* gel in paraffin oil, obtained by casting 0.2742 g of hot solution on one of the inner optical faces of the cell. Scans were performed between 240 and 600 nm, with a scan speed of 20 nm/min, a digital integration time (DIT) of 2 s, and a data pitch of 0.2 nm, averaging over four accumulations. The observed ellipticity, θ, is reported as mdeg.

### 4.5. Rheological Measurements

Both measurements carried out on *(R)*-9-HSA and *(R)*-12-HSA gels in paraffin oil (1% *w/w*) were performed within a couple of hours of preparation and after eight days of storage in a thermostat at 25 °C to prove the long-term stability.

Rheological measurements were performed using a stress-controlled rheometer (Kinexus + Ultra, Malvern, UK) fitted with a stainless-steel cone and plate geometry (40 mm diameter and angle 1°) and Peltier plate temperature control. Experimental data were collected at 25 °C by the instrumental software rSpace 1.72 (Malvern) using a gap of 1 mm. After gently transferring each sample to the lower plate with a spatula, oscillatory stress sweeps at a constant frequency of 1 Hz in the range 0.100—400 Pa were performed to determine the linear viscoelastic region. Then, frequency sweep experiments were carried out at constant stress, namely at 0.5 Pa for *(R)*-12-HSA gel samples and 0.3 Pa for the *(R)*-9-HSA ones. The consistency of the experimental data points was checked by means of the Kramers-Kronig relation [45].

The critical point, corresponding to G′/G′′ cross over point in the stress controlled amplitude sweep [42], was determined by means of the instrumental software.

The yield stress, which marks the end of the linear region, was taken at the intersection of the power laws fitted to G’ at lowest and highest stress ends of the stress controlled amplitude sweep [41,42].

### 4.6. X-Ray Structural Analysis

Data collection was performed at the X-ray diffraction beamline (XRD1) of the Elettra Synchrotron (Basovizza, Trieste Italy), with a Pilatus 2M image plate detector. Complete dataset was collected at 100 K (nitrogen stream supplied by an Oxford Cryostream 700, Oxford Cryosystems, Oxford, UK) and 293 K with a monochromatic wavelength of 0.7000 Å through the rotating crystal method. The crystal was dipped in N-paratone and mounted on the goniometer head with a nylon loop. The diffraction data were indexed, integrated, and scaled using XDS [56]. The structure was solved by direct methods using SIR2014 [57]. Fourier analysis and refinement with the full-matrix least-squares method based on *F*^2^ were performed with SHELXL-2014 [58]. Anisotropic thermal parameters were applied to all non-H atoms. Hydrogen atoms were placed at calculated positions except those of OH groups, which were detected in the Δ*F* map.

Crystal data and details of refinement: C_18_H_36_O_3_, MW = 300.47, triclinic system, space group *P*1.

At RT, *a* = 4.9300(15) Å, *b* = 9.2106(25) Å, *c* = 21.089(4) Å, α = 83.62(5)°, β = 92.20(6)°, γ = 82.38(5)°, *V* = 941.9(3) Å^3^, Z = 2, *D_c_* = 1.059 g/cm^3^, μ(Mo-Kα) = 0.069 mm^−1^, *F*(000) = 336, θ range = 0.96–28.23°. Final *R*1 = 0.0507, *wR*2 = 0.1446, *S* = 1.040 for 394 parameters and 8727 independent reflections of which 8410 with *I* > 2σ(*I*); max positive and negative peaks in Δ*F* map = 0.243, −0.160 e. Å^−3^.

At 100 K, *a* = 4.8320(10) Å, *b* = 9.1394(18) Å, *c* = 20.737(3) Å, α = 83.156(8)°, β = 89.865(9)°, γ = 82.130(9)°, *V* = 900.6(11) Å^3^, Z = 2, *D_c_* = 1.108 g/cm^3^, μ(Mo-Kα) = 0.073 mm^−1^, *F*(000) = 336, θ range = 0.99–28.70°. Final *R*1 = 0.0364, *wR*2 = 0.0968, *S* = 1.104 for 394 parameters and 8525 independent reflections of which 8496 with *I* > 2σ (*I*); max positive and negative peaks in Δ*F* map = 0.299, −0.397 e. Å^−3^.

CCDC-1480705 and CCDC-1912676 contain the supplementary crystallographic data for this paper, at 100K and RT, respectively. These data can be obtained free of charge from the Cambridge Crystallographic Data Centre, CCDC, 12 Union Road, Cambridge, CB2 1EZ, UK (fax: + 44 1233 336 033; email: deposit@ccdc.cam.ac.uk or http://www.ccdc.cam.ac.uk/deposit).

### 4.7. Synchrotron X-Ray Powder Diffraction

The powder samples of *(R)*-9-HSA crystallized in CH_3_OH, CH_3_CN, and CCl_4_ were measured at the MCX beamline [59] of Elettra Sincrotrone (Basovizza, Trieste, Italy) in transmission mode at room temperature (25 °C) with a monochromatic wavelength of 1.55 Å (8 KeV) and 1·× 0.3 mm^2^ spot size. The same beamline and parameters were used to collect a powder diffraction pattern from a sample crystallized from methanol, melted at high temperature and recrystallized by slow cooling to RT (see Section 4.2). Powder samples were loaded and packed in a 0.3 mm boron capillary, mounted on a standard goniometric head, and spun during data collection. The diffraction patterns were recorded through a scintillator detector in the 1—60° 2θ range. Diffraction data from the *(R)*-9-HSA crystalline powder obtained from paraffin oil were collected at the X-ray diffraction beamline (XRD1) of Elettra Synchrotron, Trieste (Italy) [60] in transmission mode at room temperature (25 °C), with a monochromatic wavelength of 0.700 Å (17.71 KeV) and 0.2 × 0.2 mm^2^ spot size, using a Dectris Pilatus 2M hybrid-pixel area detector. For comparison, 2theta values of this pattern were converted to match the values obtained using a wavelength of 1.55 Å.

The experimental data recorded on the powder samples obtained from methanol, acetonitrile, and carbon tetrachloride (Figure S9) resulted isomorphous, with the single crystal obtained from methanol (see simulated powder pattern in Figure S8).

In the indexing of the diffraction pattern of *(R)*-9-HSA recrystallized from melted, several possible solutions were identified using the program EXPO2014 [61]. Considering that the compound crystallized is enantiomerically pure and therefore can only pack in acentric space groups, the most reliable and meaningful solution obtained from the indexing is the one with a triclinic unit cell in the space group *P*1, with volume of 996.39(3)Å^3^, number of formula units Z = 2, and calculated density of 1.002. Refined unit cell parameters are: *a* = 4.454(1) Å, *b* = 5.02(3) Å, *c* = 49.280(1) Å, α = 89.610 (3)°, β = 89.655(5)°, γ = 64.730(3)°. The structure of this polymorph of *(R)*-9-HSA was solved by a simulated annealing protocol included in EXPO2014 using a rigid body starting from the *(R)*-9-HSA single crystal model. The final Rietveld refinement was performed with TOPAS V5 [Coelho, A. A. TOPAS-Academic, version 4.1, Coelho Software: Brisbane, Australia, 2007], and soft restrains on the atom distances (± 0.03Å) and angles (± 0.1°) were applied, resulting in a Rwp of 3.6% (Figure 8 - black curve). A Rietveld refinement using this model have been performed on the paraffin oil experimental data, resulting in a Rwp of 3.8%.

CCDC-1920339 contains the supplementary crystallographic data for *(R)*-9-HSA recrystallized from melted reported in this paper. These data can be obtained free of charge from the Cambridge Crystallographic Data Centre, CCDC, 12 Union Road, Cambridge, CB2 1EZ, UK (fax: + 44 1233 336 033; email: deposit@ccdc.cam.ac.uk or http://www.ccdc.cam.ac.uk/deposit).

## 5. Conclusions

*(R)*-9-HSA, a compound studied for its antiproliferative properties in different cancer cell lines, was analyzed from different perspectives in order to identify the structural determinants of its rheological properties. Although *(R)*-9-HSA is somewhat less efficient in inducing gelation of solvents than its positional isomer *(R)*-12-HSA, the availability of high-resolution structural data of the former can shed light on the debated molecular architecture of organogels formed by hydroxyacid of natural origin. The weak and soft gel formed by *(R)*-9-HSA in paraffin oil has a limited stability in time, different from the long-term stability of its cognate with the OH group shifted by three carbons, demonstrating that the position of the OH group heavily impacts on the organogelling ability of the molecule.

The present structural studies revealed the existence of two different polymorphs of the chiral nonracemic *(R)*-9-HSA, depending both on solvent and thermal history. The polymorph that was obtained by crystallization from polar solvents is characterized by a carboxylic acid dimer endowed with supramolecular chirality, which packs head-tail in interdigitated fashion. Hydrogen bonding involving the OH groups sitting on the midchain chiral carbon plays a paramount role in determining this kind of packing, analogous to the case of the methyl ester of *(R)*-12-HSA, which displays a quite close packing.

In the latter form, which is obtained from the melt and from the paraffin oil gel, the molecules are arranged quite differently. Actually, the XRD pattern suggests a bent dimer, which stacks in layers with different tilt.

Interestingly, both forms correspond to literature models for the packing of *(R)*-12-HSA in the organogel networks. Thus, the microstructural descriptions of this important class of low molecular weight organogelators is not unique, but depends on the molecule, the solvent and the gelation conditions. Thus, straightforward future developments of the present study will explore the influence of different solvents, temperature ramps, and other positional isomers of *(R)*-9-HSA on both supramolecular structure and organogelation properties.

## Figures and Tables

**Table 1:** Torsion angles (°) indicating the different conformation of the carboxylic groups with respect to the alkyl chains.

	CH_3_OH—Single Crystal	
	Molecule 1	Molecule 2
O1-C1-C2-C3/O5-C21-C22-C23	−21.0(3)	41.3(3)
O2-C1-C2-C3/O4-C21-C22-C23	160.4(2)	−141.2(2)
C1-C2-C3-C4/C21-C22-C23-C24	−179.12(15)	−174.25(15)

**Table 2:** Geometrical parameters of H-bonds. Atom numbering refers to Figure 6a, atoms marked with asterisks belong to symmetry-related molecules.

Donor-H	D-H	H...A	D...A	D-H...A	Acceptor
O(2)-H(2)	0.89(4) Å	1.76(4) Å	2.645(3) Å	171(4)°	O(5)
O(4)-H(4)	1.26(4) Å	1.43(4) Å	2.675(3) Å	169(4)°	O(1)
O(3)-H(3)	0.77(3) Å	2.06(3) Å	2.795(2) Å	163(3)°	O(6) *
O(6)-H(6)	0.85(3) Å	1.97(3) Å	2.791(2) Å	164(3)°	O(3) *

**Table 3:** Refined unit cell parameters for *(R)*-9-HSA crystallized from solvents (CH_3_OH, CH_3_CN, and CCl_4_) and from melted, and comparison with the unit cell parameters of the single crystal obtained from methanol.

	CH_3_OH—Single Crystal	CH_3_OH–Powder	CH_3_CN–Powder	CCl_4–_Powder	Crystallized from Melt–Powder
S.G.	*P1*	*P*1	*P*1	*P*1	*P*1
a (Å)	4.9300(15)	4.926(2)	4.982(4)	4.982(4)	4.454(1)
b	9.2106(25)	9.210(2)	9.217(4)	9.217(4)	5.02(3)
c	21.089(4)	21.084(4)	21.158(8)	21.158(8)	49.280(1)
α (°)	83.62(5)	83.62(1)	83.54(1)	83.54(2)	89.610(3)
β	92.20(6)	92.20(9)	91.93(9)	91.93(9)	89.655(5)
γ	82.38(5)	82.38(19	82.86(1)	82.86(1)	64.739(3)
V (Å^3^)	941.9(3)	941.9(1)	956.3(1)	956.3(1)	996.39(3)
d (g/cm^3^) ^1^	1.059	1.059	1.044	1.044	1.002

^1^ Calculated density.

## Supplementary Materials

The following are available online at https://www.mdpi.com/1420-3049/24/15/2854/s1, PDF file containing thermal analysis, further CD spectra and structural details.
