# Supplementary material for: X-Ray Crystal Structures and Organogelator Properties of (R)-9-Hydroxystearic Acid
Source: Molecules. 2019 Aug 6;24(15):2854. doi: 10.3390/molecules24152854 (PMC6695734; doi:10.3390/molecules24152854)
Supplement: Supplementary file 1 [file molecules-24-02854-s001.pdf]

Supplementary Material

X-Ray Crystal Structures and Organogelator Properties of *(R)*-9-Hydroxystearic Acid

Fioretta Asaro[1,*], Carla Boga[2], Nicola Demitri[3], Rita De Zorzi[1], Sara Drioli[1], Lara Gigli[3], Gabriele Micheletti[2], Patrizia Nitti[1,*], and Ennio Zangrando[1]

1 Department of Chemical and Pharmaceutical Sciences, University of Trieste, via L. Giorgieri 1, 34127 Trieste, Italy; fasaro@units.it (F.A.), rdezorzi@units.it (R.D.Z.), sdrioli@units.it (S.D.), pnitti@units.it (P.N.), ezangrando@units.it (E.Z.)

2 Department of Industrial Chemistry "Toso Montanari", University of Bologna, viale del Risorgimento 4, 40136 Bologna, Italy; carla.boga@unibo.it (C.B.), gabriele.micheletti3@unibo.it (G.M.)

3 Elettra – Sincrotrone Trieste, S.S. 14 Km 163.5 in Area Science Park, 34149 Basovizza, Trieste, Italy; nicola.demitri@elettra.eu (N.D.), lara.gigli@elettra.eu (L.G.)

Index

p. S3	Figure S1	DSC curves for *(R)*-9-HSA
p. S3	Figure S2	Picture of 1 % w/w gel samples in paraffin oil
p. S4	Figure S3	UV-visible absorption spectrum of paraffin oil
p. S4	Figure S4	CD spectra of 0.5 % w/w gels in paraffin oil, for *(R)*-9-HSA and *(R)*-12-HSA
p. S5	Figure S5	CD spectra from a freshly prepared 0.5 % w/w *(R)*-9-HSA paraffin oil gel
p. S5	Figure S6	CD spectra for 1.0 % w/w *(R)*-12-HSA gels in cyclohexane and *n*-hexane
p. S6	Figure S7	CD spectra for the gel of *(R)*-12-HSA at 0.5 % w/w in paraffin oil from a 1 cm and a 2 mm quartz cell
p. S6	Figure S8	XRD pattern for *(R)*-9-HSA at 110 K calculated from the single crystal X-ray diffraction data
p. S7	Figure S9	XRD patterns of *(R)*-9-HSA samples crystallized in CH_3OH, CH_3CN, and CCl_4
p. S7	Figure S10	XRD patterns for *(R)*-9-HSA solid from aged paraffin oil gel and the solid obtained after recrystallization from melt
p. S8	Figure S11	Molecular packing in the crystal structure of *(R)*-9-HSA after melting and recrystallization
p. S8	Table S1	Refined unit cell parameters for *(R)*-9-HSA
p. S9	Table S2	Temperature-dependent changes in torsion angles (°) of the carboxylic groups with respect to the alkyl chains
p. S9	Table S3	Temperature-dependent changes in hydrogen bond lengths in the structures of (*R*)-9-HSA crystallized from methanol, determined at 100 K and RT
p. S9	Table S4	Geometrical parameters of H-bonds in the structure of *(R)*-12-HSA methyl ester.
p. S9	Table S5	Torsion angles (°) indicating the conformation of the carboxylic group with respect to the alkyl chain for *(R)*-12-HSA methyl ester.

Differential Scanning Calorimetry

DSC measurements were performed by a Q2000 (TA instruments, US), with a temperature ramp of 5 C/min during both heating and cooling, on a sample of 4.6 mg of *(R)*-9-HSA crystallized from methanol.

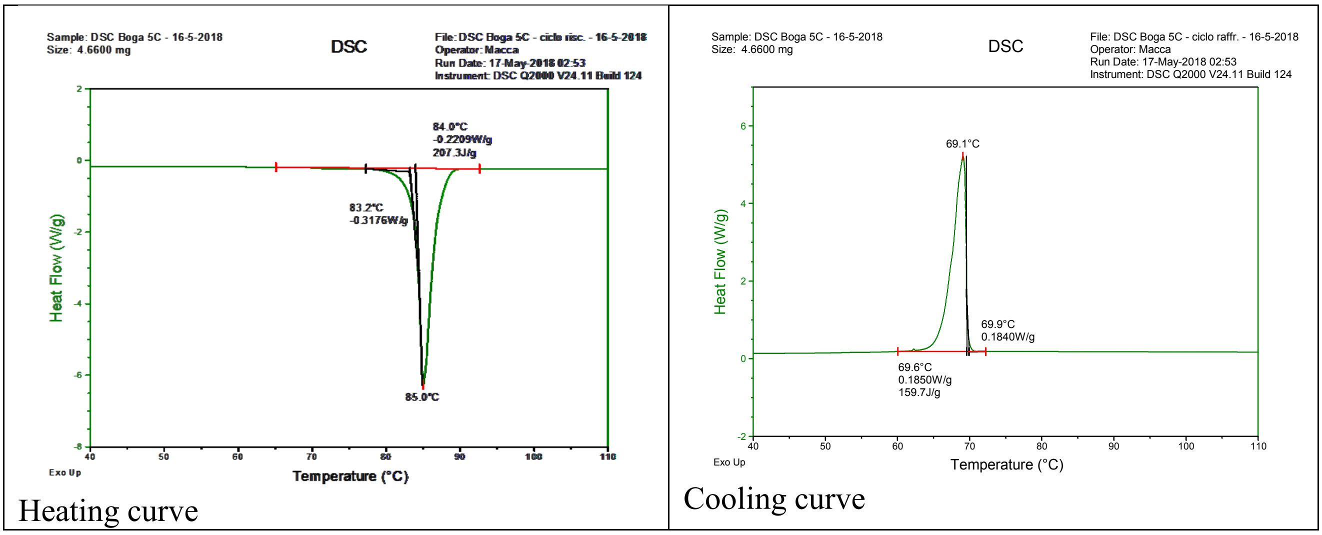

Figure S1. DSC curves for *(R)*-9-HSA.

On heating: ΔH_M= 207 J/g, $T_{onset\ M}$= 83.2 °C and T_M peak at 85.0 °C.
On cooling: ΔH_{Cr}= 160 J/g, $T_{onset\ Cr}$= 69,9 °C and T_{Cr} peak at 69.1 °C.

(R)-9- and *(R)*-12-HSA Organogels

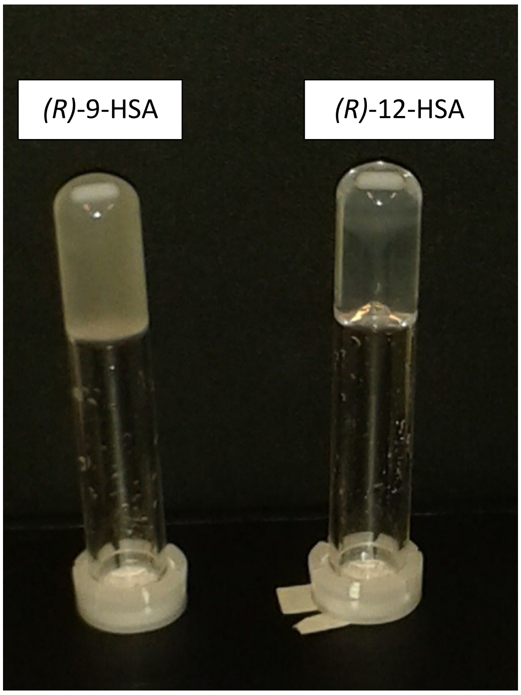

Figure S2. 1 % w/w gel samples in paraffin oil prepared by microwave irradiation. In each sample there is a small magnetic stirrer bar.

Uv-visible absorption

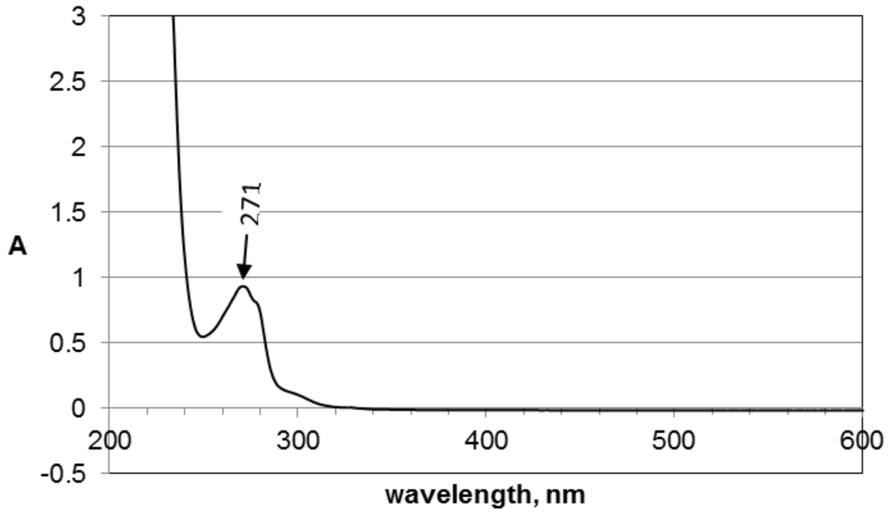

Figure S3. UV-visible absorption spectrum of paraffin oil recorded by a Shimadzu UV-2450 spectrophotometer.

Circular Dichroism Spectra

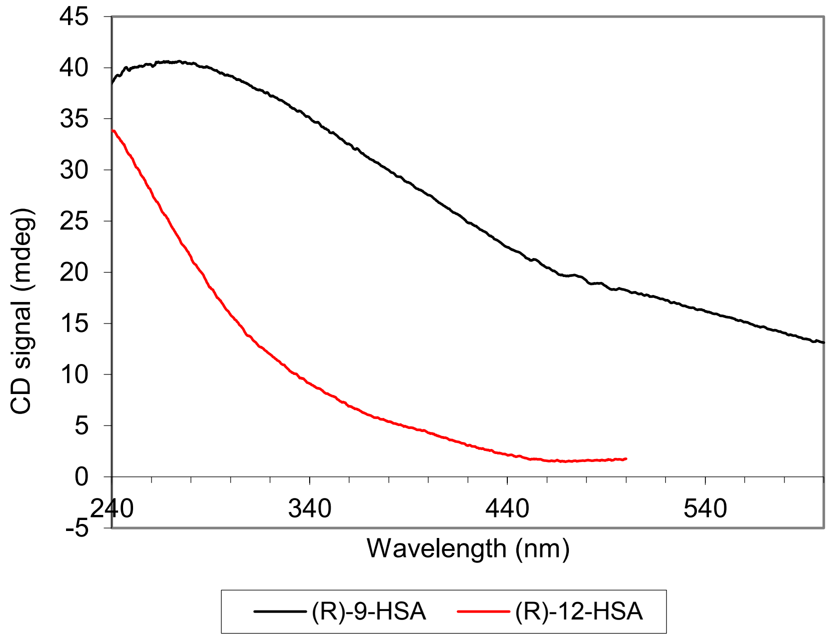

Figure S4. Comparison of the CD spectra of 0.5 % w/w gels in paraffin oil, for *(R)*-9-HSA (black) and *(R)*-12-HSA (red). The layers were obtained by casting 0.2742 g, for the former, and 0.2297 g, for the latter, of a hot solution on one of the optical faces inside a 1 cm quartz cuvette.

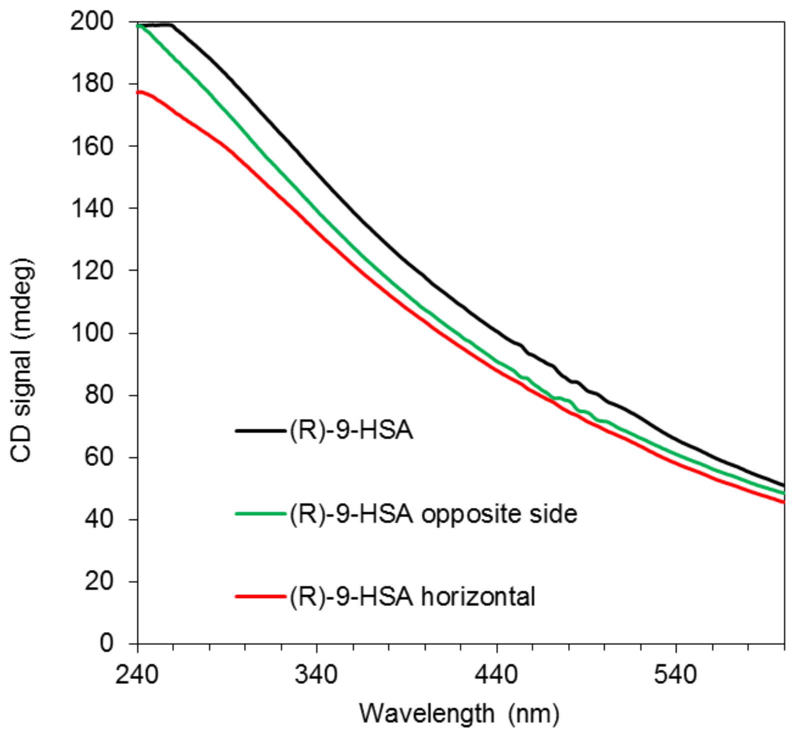

Figure S5. CD spectra from a freshly prepared 0.5 % w/w *(R)*-9-HSA paraffin oil gel sample in a 1 mm optical path quartz cell, on rotating the cell both vertically (front: black and back: green) and by 90 ° (red) to check eventual linear dichroism effects.

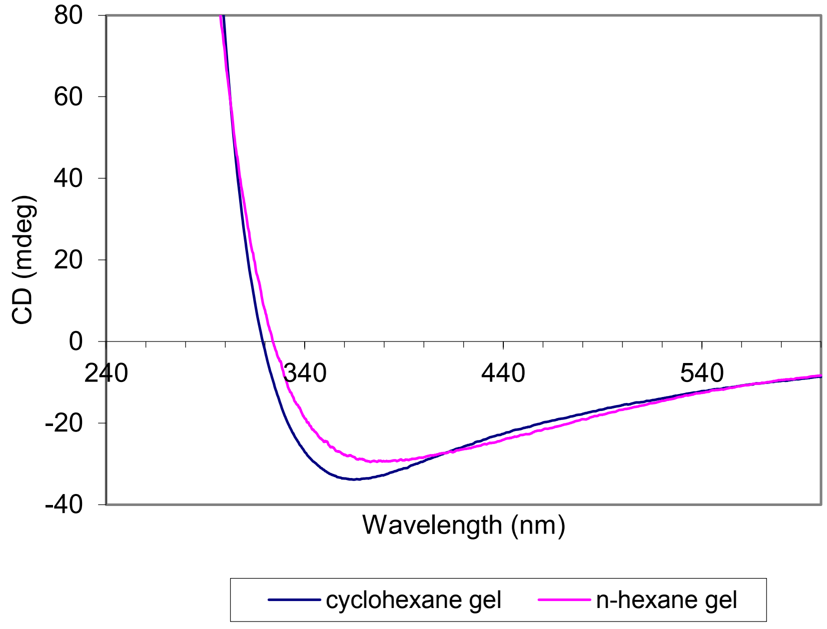

Figure S6. CD spectra for 1.0 % w/w *(R)*-12-HSA gels in cyclohexane (blue) and *n*-hexane (pink)

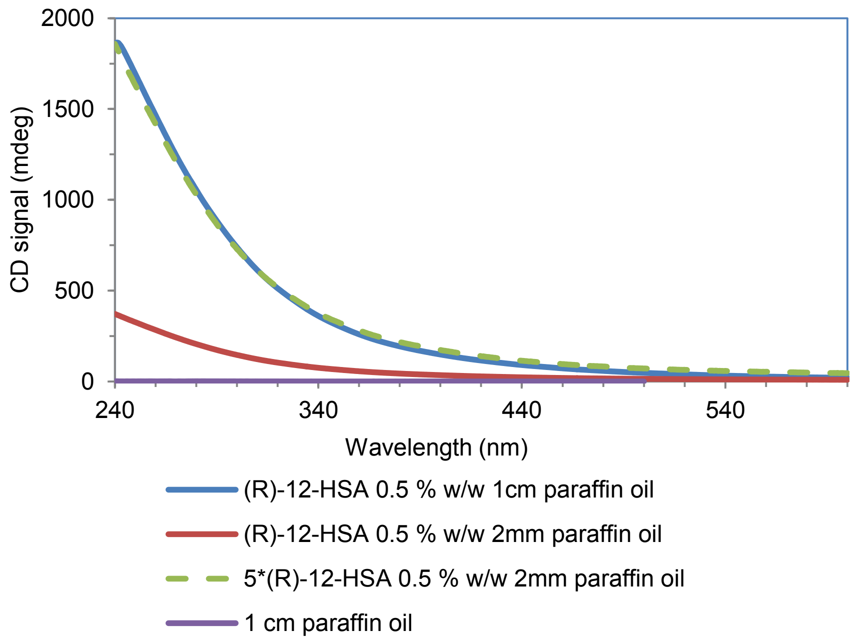

Figure S7. CD spectra for the gel of *(R)*-12-HSA at 0.5 % w/w in paraffin oil from a 1 cm quartz cell (blue), a 2 mm quartz cell (red), the latter trace multiplied by 5 (green dashed), paraffin oil (violet)

The CD spectra recorded from cells of different optical length show that the quartz surface does not affect the CD signal.

XRD Pattern of the *(R)*-9-HSA Crystal

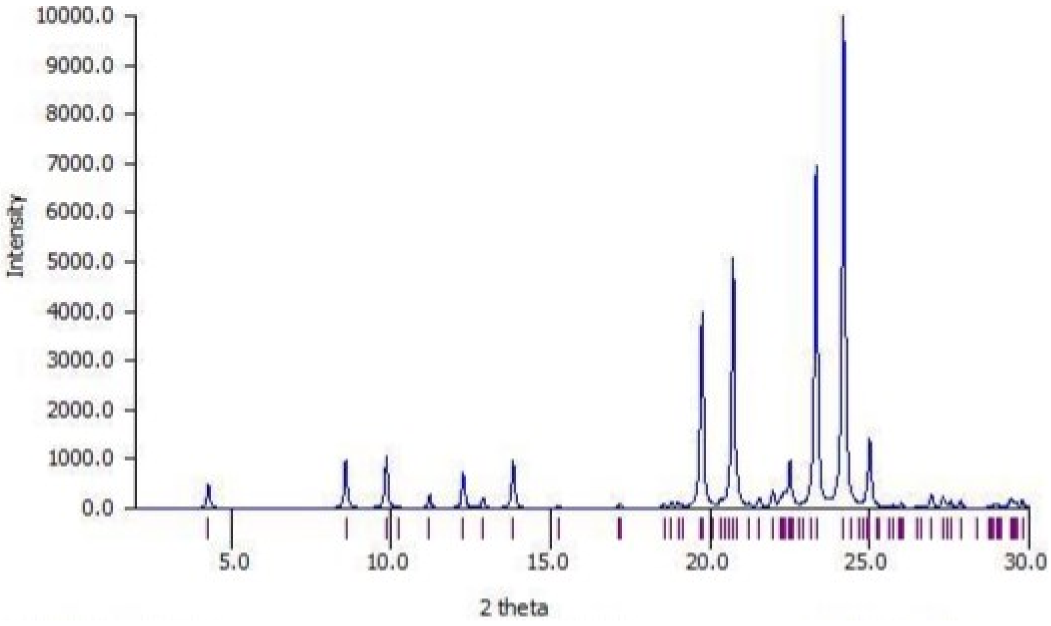

Figure S8. XRD pattern for *(R)*-9-HSA at 110 K calculated from the single crystal X-ray diffraction data for the Cu Kα radiation wavelength (1.5418 Å)

Experimental XRD Patterns

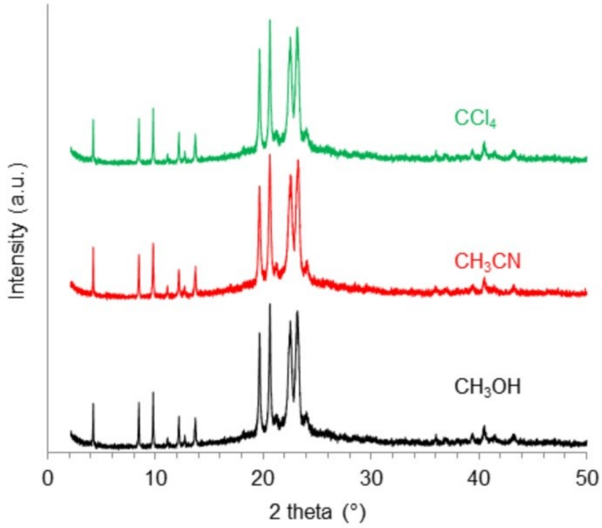

Figure S9. XRD patterns of three *(R)*-9-HSA samples crystallized in CH_3OH, CH_3CN, and CCl_4, respectively.

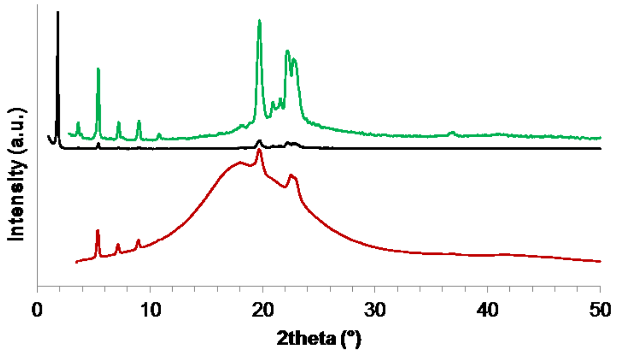

Figure S10. XRD patterns for *(R)*-9-HSA solid from aged paraffin oil gel (bottom trace dark red) and the solid obtained after recrystallization from melt (upper trace, black line, with the 5x magnification of the 2.8-50 ° region, in green).

The peaks at lowest angles, in particular the one at 1.9° 2theta, are not visible in the lowest pattern because to improve the signal to noise ratio of the sample obtained from the paraffin, it was collected at 0.7 Å at the XRD1 beamline of Elettra synchrotron characterized by a high flux and the presence of a Pilatus 2M with a very low noise read-out detector. The experimental condition allowed us to obtain a good pattern but at the same time the beam stopper masked peaks below 3° of 2theta.

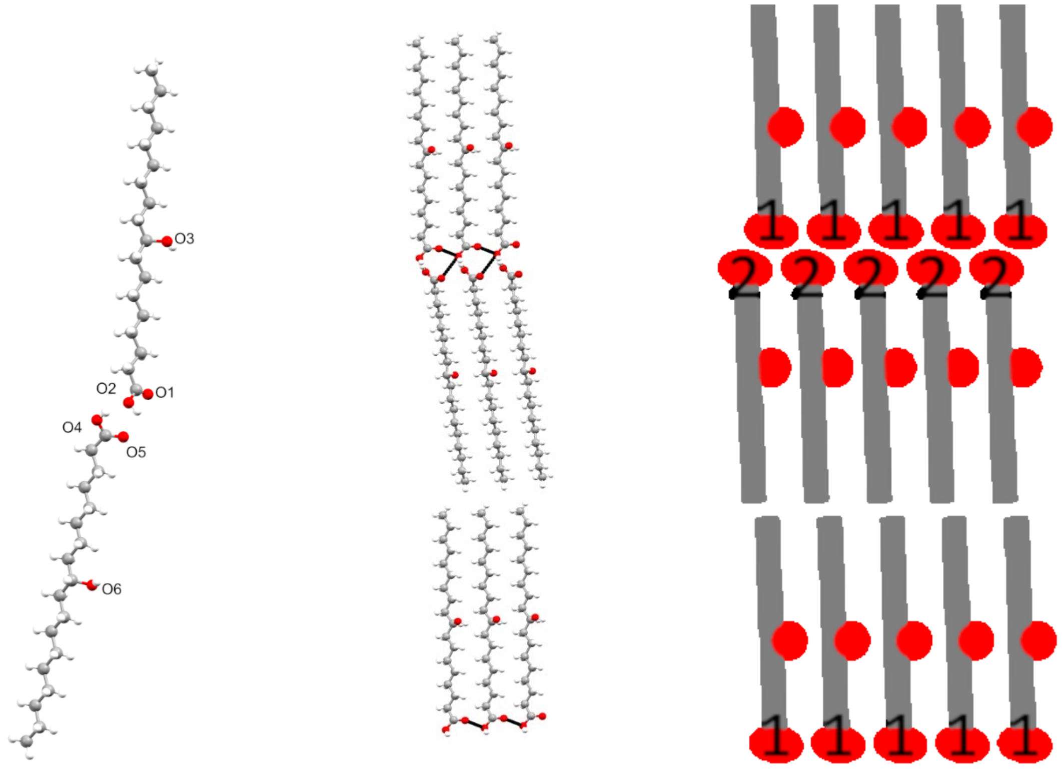

(a) (b) (c)

Figure S11. (a) The crystallographic independent unit in the crystal structure of *(R)*-9-HSA after melting and recrystallization: the *(R)*-9-HSA dimer, with molecule **1** above and molecule **2** below. **(b)** Layers of *(R)*-9-HSA molecules in the crystal packing, as viewed along the b crystallographic direction. **(c)** Schematic representation of the layer arrangement of molecules of *(R)*-9-HSA, in the same direction as **(b)**. Heads are depicted as red ovals, tails as grey rectangles, and hydroxyl groups as red circles. Numbers highlight that layers are formed by the repetition of a single crystallographically independent molecule (**1** or **2**).

X-Rays Crystal Structure Parameters for *(R)*-9-Hydroxystearic Acid

Table S1. Refined unit cell parameters for *(R)*-9-HSA crystallized from CH_3OH, CH_3CN, and CCl_4, and comparison with the unit cell parameters of the single crystals obtained from methanol.

	**100 K** [1]	**rT** [1]	**CH_3OH**	**CH_3CN**	**CCl_4**
S.G.	*P1*	*P1*	*P1*	*P1*	*P1*
a (Å)	4.832(1)	4.9300(15)	4.926(2)	4.982(4)	4.982(4)
b	9.139(1)	9.2106(25)	9.210(2)	9.217(4)	9.217(4)
c	20.737(3)	21.089(4)	21.084(4)	21.158(8)	21.158(8)
α (°)	83.156(8)	83.62(5)	83.62(1)	83.54(1)	83.54(2)
β	89.865(8)	92.20(6)	92.20(9)	91.93(9)	91.93(9)
γ	82.130(9)	82.38(5)	82.38(19	82.86(1)	82.86(1)
V ($Å^3$)	900.6(11)	941.9(3)	941.9(1)	956.3(1)	956.3(1)
d (g/cm^3) [2]	1.108	1.059	1.059	1.044	1.044

[1] Single crystal from methanol. [2] Calculated density.

Table S2. Temperature-dependent changes in torsion angles (°) of the carboxylic groups with respect to the alkyl chains.

	100 K	RT
Molecule 1		
O1-C1-C2-C3	-19.19(17)	-21.0(3)
O2-C1-C2-C3	162.00(10)	160.4(2)
C1-C2-C3-C4	-179.71(8)	-179.12(15)
Molecule 2		
O5-C21-C22-C23	40.76(17)	41.3(3)
O4-C21-C22-C23	-140.62(11)	-141.2(2)
C21-C22-C23-C24	-172.67(8)	-174.25(15)

Table S3. Temperature-dependent changes in hydrogen bond lengths in the structures of (*R*)-9-HSA crystallized from methanol, determined at 100 K and RT. Atom numbering refers to Figure 6a.

Donor-H	D-H	H...A	D...A	D-H...A	Acceptor	Symm. Code
100 K						
O(2)-H(2)	0.832(19)	1.81(2)	2.637(3)	174(2)	O(5)	[-2+x, y, -1+z]
O(4)-H(4)	0.96(2)*	1.71(2)	2.661(3)	167.6(19)	O(1)	[2+x, y, 1+z]
O(3)-H(3)	0.860(18)	1.924(19)	2.753(3)*	161.3(16)	O(6)	[1+x, y, z]
O(6)-H(6)	0.802(19)	1.957(19)	2.743(3)*	166.3(18)	O(3)	-
rT						
O(2)-H(2)	0.89(4)	1.76(4)	2.645(3)	171(4)	O(5)	-
O(1)-H(4)	1.26(4)*	1.43(4)	2.675(3)	169(4)	O(4)	-
O(3)-H(3)	0.77(3)	2.06(3)	2.795(2)*	163(3)	O(6)	[3+x, y, 1+z]
O(6)-H(6)	0.85(3)	1.97(3)	2.791(2)*	164(3)	O(3)	[-2+x, y, -1+z]

*distances most affected by temperature increase

X-Rays Crystal Structure Parameters for *(R)*-12-Hydroxystearic Acid Methyl Ester [S1]

Table S4. Geometrical parameters of H-bonds in the structure of *(R)*-12-HSA methyl ester. The acceptor atom, marked with an asterisk, belongs to symmetry-related molecule.

Donor-H	D-H	H...A	D...A	D-H...A	Acceptor
O(3)-H(3)	0.83 Å	1.93 Å	2.739 Å	164°	O(3)*

Table S5. Torsion angles (°) indicating the conformation of the carboxylic group with respect to the alkyl chain for *(R)*-12-HSA methyl ester.

	(R)-12-HSA methyl ester
O1-C1-C2-C3	8.3
O2-C1-C2-C3	-177.2
C1-C2-C3-C4	-177.1

References

[S1] Lundén, B.-M.; Löfgren, H.; Pascher, I. Accommodation of hydroxyl groups and their hydrogen bond system in a hydrocarbon matrix. *Chem. Phys. Lipids* **1977**, *20*, 263-271.
